# High-Temperature Nanoindentation of an Advanced Nano-Crystalline W/Cu Composite

**DOI:** 10.3390/nano11112951

**Published:** 2021-11-03

**Authors:** Michael Burtscher, Mingyue Zhao, Johann Kappacher, Alexander Leitner, Michael Wurmshuber, Manuel Pfeifenberger, Verena Maier-Kiener, Daniel Kiener

**Affiliations:** 1 Department of Materials Science, Chair of Materials Physics, University of Leoben, Jahnstraße 12, 8700 Leoben, Austria; zhaomingyue510@126.com (M.Z.); michael.wurmshuber@unileoben.ac.at (M.W.); daniel.kiener@unileoben.ac.at (D.K.); 2 Department of Materials Science, Chair of Physical Metallurgy and Metallic Materials, University of Leoben, Roseggerstraße 12, 8700 Leoben, Austria; johann.kappacher@unileoben.ac.at (J.K.); alexander.leitner@posteo.at (A.L.); verena.maier-kiener@unileoben.ac.at (V.M.-K.); 3 Erich Schmid Institute of Materials Science, Austrian Academy of Sciences, University of Leoben, Jahnstaße 12, 8700 Leoben, Austria; m.pfeifenberger@posteo.de

**Keywords:** W/Cu composite, nanocrystalline, high-pressure torsion, microstructure, nanoindentation

## Abstract

The applicability of nano-crystalline W/Cu composites is governed by their mechanical properties and microstructural stability at high temperatures. Therefore, mechanical and structural investigations of a high-pressure torsion deformed W/Cu nanocomposite were performed up to a temperature of 600 °C. Furthermore, the material was annealed at several temperatures for 1 h within a high-vacuum furnace to determine microstructural changes and surface effects. No significant increase of grain size, but distinct evaporation of the Cu phase accompanied by Cu pool and faceted Cu particle formation could be identified on the specimen′s surface. Additionally, high-temperature nanoindentation and strain rate jump tests were performed to investigate the materials mechanical response at elevated temperatures. Hardness and Young′s modulus decrease were noteworthy due to temperature-induced effects and slight grain growth. The strain rate sensitivity in dependent of the temperature remained constant for the investigated W/Cu composite material. Also, the activation volume of the nano-crystalline composite increased with temperature and behaved similar to coarse-grained W. The current study extends the understanding of the high-temperature behavior of nano-crystalline W/Cu composites within vacuum environments such as future fusion reactors.

## 1. Introduction

W/Cu composites are typically used as heat sinks in power electronics and construction material for high-power switches, but are also intended as a shielding material in environments exposed to radiation [1,2,3]. Thereby, the material is subjected to high local and cyclic loads due to temperature changes as well as additional irradiation or ion bombardment [2,4]. These composite materials exhibit high thermal stability concerning their microstructure combined with predictable thermal and mechanical properties. Depending on their composition, strength or ductility is limited at room temperature and, therefore, the vulnerability for cyclic damage or fatal crack propagation due to overload events is prevalent. To further increase the applicability and counteract the brittle behavior, strength, but also ductility, of the composite material must be increased [5,6]. One possibility to do so, and concurrently increase both material parameters, is to constitute a proper grain refinement [7,8,9]. This strategy was successfully applied by performing a high-pressure torsion (HPT) process on a coarse-grained (*cg*) W/Cu alloy [10]. This results in a homogenous nano-crystalline (*nc*) W/Cu composite with enhanced mechanical properties at room temperature (RT) [10].

Studies regarding nano-lamellar W/Cu multilayer coatings revealed a decomposition of the W and Cu layers to globular W grains within a Cu matrix starting at 700 °C [11]. Additionally, the formation of voids and pores on the surface of W/Cu specimens when annealed within a vacuum furnace at this temperature is reported [11]. This circumstance was further exploited to produce a nc W foam on the surface of a nc W/Cu alloy [12]. During in situ deformation experiments on Cu/TiN, Cu/W or Cu/Cr layered micro-pillars at elevated temperatures, the formation of differently shaped Cu particles along their surface were identified [13,14,15]. Their presence is explained by a shear-assisted diffusion process along with these interfaces. The Cu-multilayer composite materials exhibit stable mechanical properties with increasing temperature under compression during micro-pillar experiments [15]. Furthermore, the investigation of nc W foam yielded promising results concerning hardness and Young′s modulus [12]. Situated on the surface of shielding components, the nc W/Cu composite material is typically exposed to He^+^ implantation causing the formation of He bubbles accompanied by crack propagation constituting common irradiation damage. This may be avoided due to the introduction of open channels and pores in a foam [16,17,18,19]. In addition, this composite material provides distinct self-healing potential, as the Cu evaporates on the exposed surface and restores the protecting W foam. Ductility may be improved by adjustment of the grain size, but also through modified alloying concepts to enhance the brittle to ductile temperature or to increase the grain boundary strength [10,20]. According to density-functional theory studies on W regarding the cohesion strength between grains, the presence of Cu was found to have no significant effect on the extent of embrittlement [20]. However, this class of nc W/Cu composite material offers several application possibilities if the mechanical properties are increased and the microstructural stability is enhanced. Therefore, the present study aims to investigate the mechanical properties and microstructural stability of the nc W/Cu composite concerning exposure to elevated temperatures.

## 2. Materials and Methods

The material examined in this study constitutes a nc W-Cu composite fabricated via the HPT process. A cg W-33 wt% Cu composite was provided by Plansee SE, Reutte, Austria as a cuboid, cut from a plate with a thickness of 10 mm. This cuboid was wire-eroded to an 8 mm diameter rod, and subsequently cut into discs with a thickness of 0.9 mm. The HPT process was performed on the cg W/Cu composite discs at room temperature (RT), applying a pressure of 7.5 GPa and a rotational speed of 1.2 rpm. Sixty rotations were applied to obtain a homogeneous and saturated nc microstructure at radii ranging from 0.5 to 4 mm of the discs. At a distance of 3.65 and 3.8 mm from the center of the disc, an applied strain of about 950 ± 60 was reached [10,21]. A detailed introduction to the HPT procedure of cg W/Cu composite can be found in reference [10]. Subsequently, a heat-treatment at 300 ℃ for 1 h within a vacuum furnace (X.Tube, Xerion Berlin Laboratories® GmbH, Berlin, Germany) was performed at about 3.0 × 10^−4^ Pa. This allows for reduction of the amount of forced mechanical intermixing between W and Cu during HPT process without resulting in significant grain coarsening or recrystallization [10,22]. Hence, the mean grain size of W and Cu grains was determined to 14.4 ± 6.3 nm in diameter [1]. This ranges in the same regime as the as-deformed specimen, exhibiting a grain size of 5–15 nm [10]. Therefore, only a minor influence of the heat treatment and, thus, testing at temperatures up to 300 °C, are suggested. Representative images showing the nanostructure of the investigated W/Cu composite can be found in ref. [12].

Subsequently, heat-treatments at 400, 500, 600, 700 and 900 °C for 1 h within a high-vacuum furnace were conducted on separate specimens to determine the temperature stability of the nc W/Cu microstructure. The specimens were previously mirror-polished to ensure an appropriate surface condition for nanoindentation tests, as well as to disclose the effects on the surface. Subsequently, a scanning electron microscope (SEM, LEO1525, Carl Zeiss GmbH, Oberkochen, Germany) was used to investigate the microstructure and surface conditions of the heat-treated specimens. Here, an acceleration voltage of 15 kV, typical magnifications ranging from 2000 to 30,000 times, working distances between 3.5 and 5.5 mm and an image resolution of 1024 × 768 pixel were used as imaging parameters. To achieve a more detailed understanding of surface effects, cross-sections were excavated by use of a Zeiss Auriga Laser FIB system containing a focused ion beam column (Orsay Physics Ga^+^ ion FIB) and a scanning electron column (Gemini Schottky field emission, Carl Zeiss GmbH, Oberkochen, Germany). To realize extended cross-sections, a femtosecond laser from type Origami 10 XP (Onefive GmbH, Zürich, Switzerland) and a pulse duration of about 500 fs was used [23]. Further polishing was performed by the use of the attached FIB device using ion cutting currents from 2 nA to 200 pA.

The RT and nanoindentation experiments up to 400 °C were performed on a G200 platform (KLA Corporation, Milpitas, CA, USA). This setup, including a continuous stiffness measurement (CSM) unit, enables the determination of hardness and Young′s modulus continuously throughout the indentation depth. A diamond Berkovich tip (Synton MDP, Nidau, Switzerland) exhibiting a tip radius of 250 nm was calibrated following the Oliver-Pharr procedure and the indentation strain rate ($\dot{P}$/*P*) was kept constant at 0.05 s^−1^ during the measurement [24,25]. A maximum load of 700 mN at an indentation depth of 2500 nm was applied during the measurements. The resultant hardness values were determined and averaged within a range of 300 to 425 nm as well as 1200 and 1400 nm. Furthermore, strain rate jump tests were performed at indentation depths of 500 and 1500 nm with respective constant indentation strain rates of 0.01 and 0.005 s^−1^ up to a total indentation depth of 2500 nm [26]. To avoid oxidation during the experiments, a protective gas atmosphere was applied including a constant gas flux of 0.4 to 0.6 L/min.

In the case of HT nanoindentation tests from 400 to 600 °C, an InSEM-HT device (Nanomechanics Inc. KLA, Oak Ridge, TN, USA) including a CSM module was utilized. To ensure stable temperature conditions and guarantee an isothermal contact between tip and specimen, both are heated and controlled separately [27]. To avoid specimen oxidation or degradation of the indenter tip at high testing temperatures, the system is mounted within a Tescan Vega3 SEM (Tescan, Brno, Czech Republic) and is operated under high-vacuum conditions [28]. The used Berkovich type indenter tip consists of silicon carbide (Synton-MDP, Nidau, Switzerland). The machine compliance and area function calibration of the tip were determined by indentation tests on fused silica at RT [25]. Possible degradation of the tip was monitored by regular indentations on fused silica. A maximum indentation depth of 600 nm and maximum force of 50 mN were utilized. Strain rate jump tests were performed with a constant indentation strain rate of 0.4 s^−1^ to a displacement of 300 nm, followed by a 0.04 s^−1^ jump for 125 nm, and a subsequent return to 0.4 s^−1^. Following Maier et al. [26], the strain rate sensitivity (*m*) and the apparent activation volume (v*) were determined.

Notably, high-temperature nanoindentation tests were performed from RT up to 600 °C on the same HPT deformed and subsequently polished specimen with both testing setups. Here, similar distances from the center point were selected to ensure comparable deformation ratios and consequently, the corresponding identic microstructure. The exact positions are visible in Figure S1a and are located directly next to each other.

## 3. Results

### 3.1. Microstructural Development

**Figure 1:**
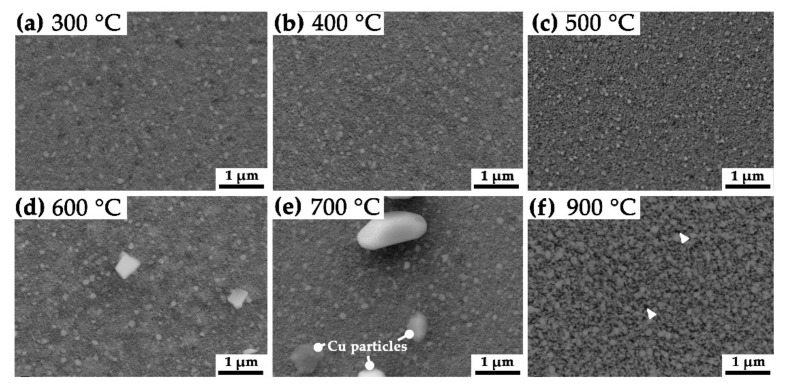
SE SEM images of the HPT processed W/Cu specimens after 1 h annealing at temperatures of (**a**) 300, (**b**) 400, (**c**) 500, (**d**) 600, (**e**) 700 and (**f**) 900 °C. Within (**d**,**e**) faceted Cu particles become visible on the polished surfaces. In (**f**) the formation of pores along the surface is visible and marked by white arrows.

Secondary electron (SE) SEM images of the HPT deformed specimens′ surface after the heat-treatments are displayed in Figure 1a–f. The specimens were heated to 300, 400, 500, 600, 700 and 900 °C for 1 h and subsequently cooled to RT within a vacuum furnace. The W grain size is slightly increased after annealing at 400 °C compared to 300 °C, as can be seen in Figure 1a,b. Upon further increasing the temperature, Cu droplets and pools are emerging on the surface of the specimen within a temperature range of 600 to 700 °C. Their number and size increase with rising temperature Figure 1d,e. Specimens annealed at 900 °C show again a homogenous surface structure without any Cu particles or pools visible in Figure 1f. However, the formation of dimples and pores on the surface indicates the evaporation of Cu from the surface. Due to the rough surface, a higher contrast between the nc W grains and evolving pores is evoked in SE imaging mode.

**Figure 2:**
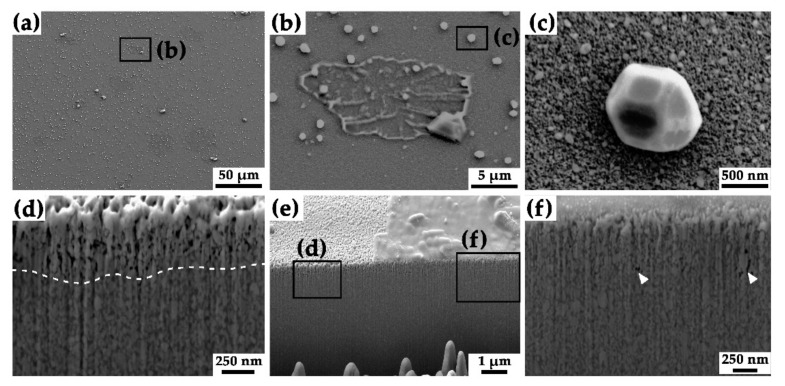
SE SEM images of a W/Cu composite annealed at 700 °C: (**a**) overview of the polished surface, (**b**) representative Cu pool, (**c**) faceted Cu particle, (**d**) sub-surface region not covered by a Cu pool, (**e**) overview of the cross-section containing different areas and sub-surface structures and (**f**) sub-surface region below the Cu pool. In (**d**,**f**) the dotted line restricts the porous zone and white arrows indicate single pores, respectively.

To further investigate the effect of Cu pools located on the surface of the respective specimen, a cross-section was realized using FIB. In Figure 2a several Cu pools are visible on the surface of the specimen heat-treated at 700 °C. Additionally, several faceted Cu particles are situated next to and atop a selected Cu pool in Figure 2b. A more detailed SE image of one of the faceted Cu particles is displayed in Figure 2c. The exposed edge of a Cu pool including two different regions below the surface is visible in Figure 2e. The cross-section reveals a shallow appearance of the Cu pools with no noticeable in-depth effects on the microstructure.

However, below the pool a lower porosity was determined (see Figure 2f) compared to the uncovered material in Figure 2d. Here, the mean depth of an open porosity was determined to be 330 nm. The Cu pool covered area solely contains some loose regions including visible pores. The mean diameter of about 50 Cu pool determined from SEM images at 700 °C was 18.3 ± 6.7 µm. The vertical contrast visible along the cross-sections in Figure 2d,f can be attributed to curtaining effects originating from FIB preparation. Due to the re-deposition of the sputtered material on the specimen′s surface, the top layer near the edge visible in Figure 2d,f appears to exhibit a coarser appearance of the surface grains.

### 3.2. Nanoindentation and HT Nanoindentation Tests

**Figure 3:**
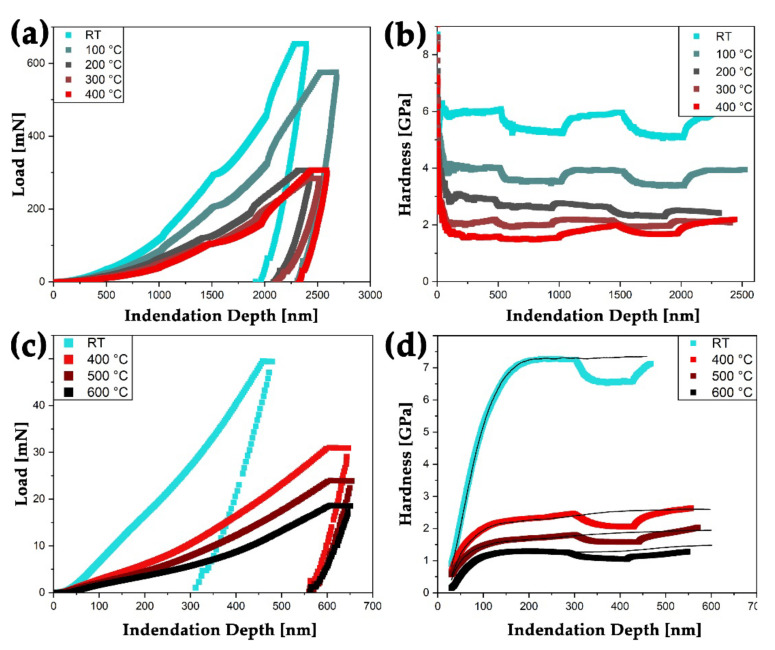
Representative indentation data of nc W/Cu specimen from indentation experiments gathered by the G200 setup (**a**,**b**) and the InSEM-HT nanoindentation testing setup(**c**,**d**). In (**a**) typical load and in (**c**) hardness vs. indentation depth curves at RT, 100, 200, 300 and 400 °C are displayed. In (**c**) load and (**d**) hardness values are displayed over indentation depth for testing temperatures at RT, 400, 500, and 600 °C, respectively. Within an indentation depth of 300 to 425 nm the constant strain rate was decreased from 0.4 to 0.04 s^−1^ for strain rate jump tests at the same temperatures. Additional indentation experiments with a constant strain rate were performed for comparison and are displayed as black lines in (**d**). For a colored representation, the reader is referred to the web version of this article.

Selected nanoindentation results of the G200 setup at RT, 100, 200, 300 and 400 °C are displayed in Figure 3. Representative load-indentation depth curves including strain rate jumps are displayed in Figure 3a. The corresponding hardness values as a function of the indentation depth are displayed in Figure 3b. Considering RT data, the force limit was hit; therefore, the aimed maximum indentation depth of 2500 nm was not reached. To compare measurements from different temperatures and later the InSEM-HT testing setup, values were determined in a depth of 300 to 450 nm, where the hardness remains almost constant.

Furthermore, representative load-indentation depth curves from the InSEM-HT setup at RT, 400, 500 and 600 °C up to either a maximal indentation depth of 600 nm or a load of 50 mN are shown in Figure 3c Their corresponding hardness values are displayed in Figure 3d and were determined with a $\dot{P}/P$ of 0.4 s^−1^. Continuous mechanical data is available due to the used CSM testing setup [26]. Additionally, hardness profiles determined from strain rate jump tests are displayed in Figure 3d. To compare different data points at varying temperatures and also to the G200 setup, mean values from 300 to 450 nm were used. Also, the associated Young′s modulus was determined within this indentation depth [29]. Here, initial surface and contact effects can be excluded, resulting in almost constant values. As expected for nc materials, no indentation size-effect was identified with increasing indentation depth [30].

**Figure 4:**
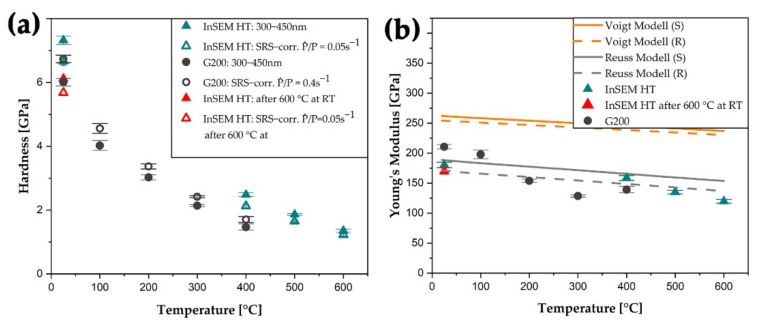
In (**a**) the evolution of the hardness as a function of the temperature of the nc W/Cu composite material is displayed. The data include a standard deviation calculated from at least five indents and was corrected according to the given constant indentation strain rate. The Young′s modulus as a function of the temperature is shown in (**b**). Guidelines based on literature data were included to guide the reader′s eyes [31,32]. Therefore, the anisotropic Young′s modulus of Cu was calculated after Shtrikman (S) and Reuss (R) bounds. The open symbols constitute strain-rate-corrected data points, and the red triangles display data points recorded subsequently to the high-temperature experiments at RT. Here, only a minor deviation of 16 and 5% from the hardness and Young′s modulus was determined, respectively. For a colored version of this article, the reader is referred to the online edition.

For comparison, the hardness and Young′s modulus values determined from different testing setups and temperatures are summarized in Figure 4. As the material is strain rate sensitive and two different constant indentation strain rates were used, the evaluated hardness from the InSEM-HT device is higher compared to the values from the G200 setup. Thus, to obtain comparable hardness values, a correction of the constant strain rate of the respective test setup was performed: values from the InSEM-HT device and G200 setup were corrected to a $\dot{P}/P$ of 0.05 and 0.4 s^−1^, which correspond to the open symbols in Figure 4a. However, at RT and 400 °C, mean deviations of 0.58 and 0.78 GPa between the InSEM-HT and corrected G200 values (open circles) were determined. Hence, hardness values from the InSEM-HT indenter remain slightly elevated compared to data gathered using the G200 setup.

The measured Young′s moduli including some trend lines calculated from literature data are shown in Figure 4b. Hence, Young′s moduli of Cu and W from Chang et al. [31] and Lowrie et al. [32] with a phase ratio of 49/51 were used. With increasing temperature, the measured Young′s modulus decreases and shows good accordance with the trend lines calculated after the Reuss model (R). Here, RT Young′s moduli from InSEM-HT setup are slightly under- and in the case of 400 °C measurements overestimate values from G200 setup, but in general, nicely follow literature predictions [31,32].

## 4. Discussion

The microstructural investigation (Figure 1) confirmed that the surface structure of heat-treated nc W/Cu composite material witnesses a distinct change of surface characteristics at varying temperatures. With increasing temperature, the nc Cu matrix phase tends to sublimate into the high-vacuum oven atmosphere. Furthermore, Cu pool-like structures are observed on the surface starting at temperatures of 600 °C. Their number and size rise with increasing temperature but vanish at 900 °C. As the temperatures increase, an accumulation of Cu pool could be determined with increasing distance from the center of the HPT deformed specimen. This indicates an influence of the applied deformation strain, as this effect amplifies with increasing distance from the center. By raising the deformation ratio, the grain size, mechanical intermixing, homogeneity but also internal stresses are enhanced and ultimately reach a saturation level [10]. Mechanical intermixing and stresses were reduced during the preliminary heat-treatment at 300 °C for 1 h within a vacuum furnace. In this condition, the mean grain size of W and Cu grains was determined to 14.4 ± 6.3 nm. In the following, no visible changes on the surface or microstructure could be determined during SE SEM investigations. Post-indentation images of the specimen′s surface indicate an additional accumulation of Cu pool in and around the area where the indentation tests were performed (see supplementary SP-1). This substantiates a stress-assisted diffusion of Cu along different interfaces, as previously observed during micropillar experiments on heat-treated Cu/TiN multilayers by R. Raghavan et al. [13]. The authors identified faceted Cu particles on the surface of tested pillars and Cu infiltrated pores along with deformation-related shear bands [13]. In our study, faceted Cu particles were identified on the specimen′s surface by SEM investigations starting at temperatures of 600 °C. Similar to the Cu pool, their number and size increase with increasing temperature and they vanish during a heat-treatment of 900 °C for 1 h within a vacuum furnace. Studies of W/Cu multilayer specimens indicate a correlation of cracks along W grain boundaries and the presence of faceted Cu particles on the surface [11,33]. Therefore, a coupled mechanism of preferred diffusion pathways and stress-induced diffusion of Cu to the surface was postulated [11,13]. As the presence of faceted Cu particles is randomly distributed on the respective surfaces visible in Figure 1d,e, a site unspecific availability of diffusion pathways within the nc microstructure is assumed. The presence and morphology of the Cu pool were further investigated from a cross-section of the specimen heat-treated at 700 °C (Figure 2). Here, the area below the surface, which is not covered by a Cu pool, exhibits a pronounced porosity up to a mean depth of 330 nm, assuming an effective loss of 70% of Cu up to this depth under consideration of the prevalent volume share of 49 vol.% Cu, theoretically 0.62 faceted Cu particles with a mean diameter of 716 nm should be present per µm^2^ on the surface of the specimen. This fraction would be equivalent to a mean coverage of 25.0% of the available surface area by Cu particles. The mean particle diameter of 716 ± 153 nm was determined from high-resolution SEM images analysis. However, the determination of SE SEM images of this specimen state yields a mean coverage from 4.3 to 5.2% of faceted Cu particles besides the Cu pools. This observation indicates a strong loss of Cu into the vacuum due to sublimation from the specimen′s surface. Similar effects were observed in the case of ZnCu alloys, as the Zn starts to evaporate at temperatures of 450 °C, and the remaining Cu forms a microporous foam [34]. This effect was described as physical vacuum dealloying, as the vapor pressure of the volatile phase is by far higher compared to the Cu phase. In the case of the nc W/Cu composite, the vapor pressure of Cu at 700 °C is more than 27 magnitudes higher compared to that of the W phase [35]. Hence, at this temperature, the vacuum pressure of Cu amounts to 5.0 × 10^−7^ Pa and ranges, therefore, below typical high-vacuum pressures of 10^−4^ to 10^−6^ Pa. This enables the deposition of Cu on the surface as Cu pools and later as faceted Cu particles due to the stress-induced diffusion mechanism. At a temperature of 900 °C, the Cu vacuum pressure increases to 5.8 × 10^−4^ Pa, resulting in the sublimation of Cu from the surface [35]. Here, the limiting factor is the replenishment of Cu via diffusional processes along or through the depleted nano-porous W-rich zone.

Underneath the Cu pool in the cross-section view (see Figure 2f) by themselves, a few agglomerated pores are visible. This indicates an easier diffusion pathway or stronger driving forces to relocate Cu from deeper regions of the specimen. Therefore, the presence of microcracks is assumed to promote the formation of Cu pools on the surface. Furthermore, faceted Cu particles are also located on these Cu pool, indicating no or at least a minor influence on the distribution and size of the pools.

The faceted morphology of the Cu particles is suggested to rely on the fact that the material is striving to achieve an optimal shape to reduce its total surface energy [36]. A visualization of this, named the Wulff plot, resembles the shape of faceted Cu nanoparticles for face-centered cubic (fcc) specimens [37]. Their shape was proven to change as the formation of specific Miller planes is favorable. under different atmospheric conditions [38,39,40]. However, the morphology of the nc W grains on the surface of the heat-treated specimens was not noticeably changed, due to the annealing process at different temperatures, which was due to the low diffusivity of W in this temperature regime.

The results from the RT and high-temperature nanoindentation experiments must be regarded in the light of developing surface porosity and thus, changing microstructure. As the Cu phase sublimates to a larger extent with increasing temperature and time, an increasing number of open channels and pores are prevalent in the near-surface layer of the nc W/Cu composite material. As the indentation experiments were performed up to an indentation depth of 600 or 2500 nm in the case of InSEM-HT or G200 testing setup, respectively, and the respective hardness and Young′s modulus were determined within a range of 300 to 450 nm, only minor influences of the evolving porosity and other microstructural changes are assumed up to a temperature of 400 °C. This is further supported by the hardness determination of the G200 experiments within a depth of 1200 to 1400 nm, resulting in comparable values within the given standard deviation. Also, hardness and modulus are deduced from the plastic and elastic deformation regions, which reach to a depth of around 5–15 times deeper than the actual indentation depth. Hence, the hardness is almost constant for indentation depths exceeding 200 nm at any temperature. Furthermore, after strain rate jump tests and returning to the initial strain rate, similar hardness values were reached with both used test setups (see Figure 4a). In a study on a nc W foam with a similar initial grain size but higher deformation applied using a severe plastic deformation process, lower hardness values of the pure foam of 2.8 GPa were determined at RT [12]. Furthermore, the minor influence of pores on the surface of nc W/Cu composite is substantiated by elevated hardness values determined from the InSEM-HT test setup at RT after the performed G200 experiments (see Figure 4a). Here, the strain rate corrected hardness from the InSEM-HT device exceeds the G200 RT hardness by 0.66 GPa and amounts to 6.67 ± 0.13 GPa. At the given temperature, this value is similar to hardness values of cg W specimens investigated by Kappacher et al. [41]. Furthermore, there is a good consistency of hardness and Young′s modulus values with deposited and heat-treated nc W-Cu thin films [22,29,42]. With increasing temperature, the hardness of the nc W/Cu composite decreases steadily. This behavior may be attributed to the drastic drop in flow-stress of body-centered cubic (bcc) materials with increasing temperature [41,43]. Hence, the thermal activation of kink-pairs along the ½ <111> screw dislocation lines at a temperature of about 0.2 T_m_ enables plastic deformation under applied shear stresses [44]. Above this temperature, the athermal regime is governed by both, edge and screw, dislocation processes which are independent of thermal activation [41,44]. Since the drop in hardness with increasing temperature in the here investigated nc W/Cu is even more pronounced compared to the cg W, a major influence of the Cu phase is suggested. Moreover, the critical temperature of 0.2 T_m_ of W was not reached during the in situ nanoindentation experiments. Different studies are proposing a hardness between 9 and 11 GPa for nc Cu at RT, with a strong influence of the grain size and a Hall-Petch breakdown below a grain size of 7 to 10 nm [45,46,47]. However, the specimen′s mean grain size after the heat-treatment at 300 °C for 1 h amounts to 14.4 ± 6.3 nm for the W and Cu grains. This is in the same range as the as-deformed HPT processed W/Cu [10]. Therefore, a minor effect of the heat-treatment and testing temperatures up to 300 °C on the initial microstructure is assumed.

Young′s modulus also decreases with increasing temperature. To guide the reader′s eyes, different trendlines were included in Figure 4b. They were calculated including their phase composition according to the Voigt and Reuss model [31,32,48,49]. In the case of the strong anisotropic Young′s modulus of Cu, methods after Shtrikman (S) and Reuss (R) were implemented [31,50,51]. Hence, the best accordance was achieved with the Reuss model (R), visible in Figure 4b, representing the lowest bound of Cu, which was adapted to the temperature [50]. Under consideration of the InSEM-HT data points (green triangles in Figure 4b), a deviation of the linear behavior at temperatures of 500 and 600 °C of the two different models can be evidenced. This drop in Young′s modulus amounts to about 17 or 26 GPa at temperatures of 500 and 600 °C, respectively. After the performed G200 and InSEM-HT measurements, a reference measurement was conducted at RT, depicted as the red triangle in Figure 4b. Here, a Young′s modulus reduction of 10 GPa indicates a minor change of testing conditions due to the formation of pores along the surface, a distinct evaporation of Cu or minor grain growth after heating to a temperature of 600 °C.

The strain rate sensitivity (*m*) and activation volume (v*) were calculated using strain rate jump tests during indentation experiments according to the method of Maier et al. [26]. Typically, *m* is determined as a function of grain size—leading to a decreased *m* value with decreasing grain size for bcc materials at RT [52,53,54,55]. In the case of fcc crystals, this trend is reversed, as the governing mechanism turns from cutting of forest dislocations in coarse grains to a dislocation-grain boundary interaction along nc grains [53,56,57].

As demonstrated for ultra-fine grained Cr, *m* increases above the critical temperature of 0.2 T_m_. This fact is attributed to a mechanism change when the critical temperature is reached and fcc-like behavior governs the plastic processes along with grain boundary contributions [44]. In more detail, thermally activated grain boundary-dislocation interactions become dominant against the contribution of screw dislocation movement by kink-pair interactions [44,54]. This was also determined with un- and deformed W, where *m* increases in the deformed state with increasing temperature [55,58]. In general, fcc materials exhibit lower *m* values compared to bcc materials, as Peierl′s stress is lower and, therefore, the effect of temperature and further time-dependent influences are not as pronounced. However, especially in ufg or nc conditions, an increasing *m* with increasing temperature is reported [59].

**Figure 5:**
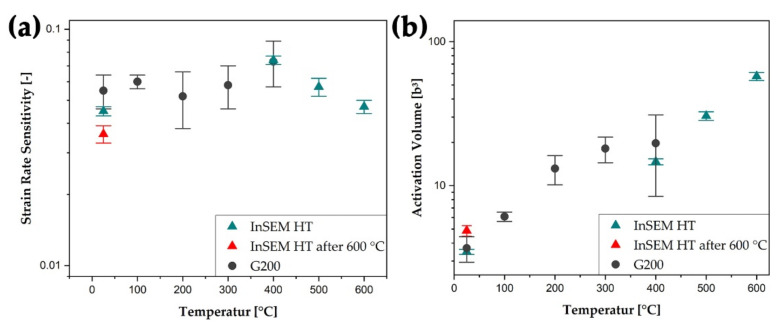
In (**a**) the strain rate sensitivity and (**b**) the calculated activation volume of the nc W/Cu composite are plotted as a function of testing temperature. The activation volume was normalized to the cubed Burgers vector (b^3^) to enable comparison between different material systems [26,28]. The round markers indicate G200 and the triangular ones the InSEM-HT data points, respectively. Red triangles depict measurements at RT, which were performed after heating and testing up to a temperature of 600 °C. For a colored version of this article, the reader is referred to the online version.

In the case of nc W/Cu composites, differently pronounced and mutually overlapping effects are affecting the temperature-dependent condition of *m*. Since the critical temperature of W is typically reached at temperatures above 600 °C, an increase of *m* due to the thermally activated grain boundary-dislocation interaction mechanism can be neglected [58]. Furthermore, a constant influence of *nc* W grains on *m* is suspected within the tested temperature range [28]. The literature unveils a strong increase of *m* with increasing temperature within *nc* Cu, but is solely available up to a temperature of 300 °C [59,60]. The determined strain rate sensitivity of the nc W/Cu composite increases slightly with increasing temperature up to 400 °C (see Figure 5a). However, no clear trend can be evaluated, as all values range within the measured standard deviation. At temperatures from 400 to 600 °C, a decrease of *m* was determined from the InSEM-HT data. This may be attributed to the decreasing influence of the Cu phase, since a distinct amount of Cu was evaporated. To verify the measurement, a second RT test was performed and a strain rate sensitivity decrease of about 0.01 was determined. This deviation represents the drop in *m* from 500 to 600 °C, substantiating the effect of pores along with the surface layer.

As can be seen in Figure 5b, the calculated v* increases from ~3.6 b^3^ at RT to about 57 b^3^ at 600 °C. A similar trend was reported by Kappacher et al. regarding the activation volume of cg W [41]. However, measured RT values in the present study are below *nc* or *ufg* representatives from the literature, wherein, typically, values around 10 b^3^ were determined for both, *fcc* and *bcc* materials [28,61,62]. This suggests an even lower grain size for the *nc* W/Cu composite. Contrary to this, with increasing temperature, a steady rise of v* was determined. This agrees well with the literature, where stabilisation of v* above 0.2 T_m_ could be determined after rising values for ufg W and Cr [28,62]. In the present work, the regarded unit cell was defined as the W cell, since the W grain size stays almost constant and no drastic evaporation effects could be determined for this element. In addition to the increasing temperature, the decreasing hardness further results in higher v* values at elevated temperatures. Very limited literature data is available for the mechanical properties of *nc* or ufg materials at temperatures as high as 600 °C. Also, the influence of the 300 °C annealing heat-treatment may influence the number of preexisting dislocations, thus, affecting the mechanical behavior of the material up to the respective annealing temperature. However, the increasing activation volume indicates a strong influence of the W phase on the deformation behavior. Another aspect may be the coarsening of Cu grains, forming a continuous matrix phase around the nc W grains. This, also, would consequently result in an increased v*, as longer pinned dislocation segments become possible.

## 5. Conclusions

Within this study, a nano-crystalline W/Cu composite processed via the HPT process was investigated regarding the microstructure and mechanical properties at elevated temperatures. The material was heat-treated in a vacuum furnace at various temperatures to achieve information about the stability of each phase as well as the grain size during annealing. High-temperature nanoindentation allowed for the quantification of hardness and Young′s modulus of the material in situ between room temperature and 600 °C. Furthermore, strain rate jump tests were performed to calculate *m* and the related v* and assess the underlying deformation mechanisms.

Scanning electron microscope investigations on the heat-treated specimens unveiled only minor changes of the microstructure up to 600 °C. At 900 °C, a distinct coarsening of W and Cu grains was evident. On the polished surface of these specimens, the formation of pores, faceted Cu particles and Cu pools was determined. Owing to the higher vapor pressure, Cu is more prone to evaporate into the high vacuum chamber of the furnace or nanoindentation device compared to W. However, with increasing temperature, the number and size of Cu pools, as well as faceted Cu particles, increases. At a temperature of 700 °C, the porous layer exhibits a thickness of about 330 nm. Below the Cu pools, fewer pores were observed since the Cu supply is enabled by deformation-induced diffusion along open Cu channels from deeper regions.

With increasing temperature, the hardness and Young′s modulus decrease continuously. This behavior is reasoned by the temperature-dependent motion of dislocation; therefore, easier deformability is achieved at higher temperatures. Concerning the strain rate sensitivity with increasing temperature, overlapping effects lead to a constant value within the measurement uncertainty. At the same time, the activation volume increases with temperature and thus behaves similar to the high-temperature behavior of coarse-grained W.

Based on the results of this investigation, a deeper understanding of the high-vacuum behavior and temperature dependence of nano-crystalline W/Cu composites could be gathered. With complementary nanoindentation investigations, the mechanical response at elevated temperatures was determined in regard of the present microstructure.

## Data Availability

The data presented in this study are available on reasonable request from the corresponding author.

## Supplementary Materials

The Figure S1 is available online at https://www.mdpi.com/article/10.3390/nano11112951/s1, Figure S1: Cu pools.

## References

[1] Zhao M., Issa I., Pfeifenberger M.J., Wurmshuber M., Kiener D. (2020). Tailoring ultra-strong nanocrystalline tungsten nanofoams by reverse phase dissolution. Acta Mater..

[2] Rieth M., Dudarev S.L., Gonzalez de Vicente S.M., Aktaa J., Ahlgren T., Antusch S., Armstrong D., Balden M., Baluc N., Barthe M.-F. (2013). Recent progress in research on tungsten materials for nuclear fusion applications in Europe. J. Nucl. Mater..

[3] Chen S., Bourham M., Rabiei A. (2015). Neutrons attenuation on composite metal foams and hybrid open-cell Al foam. Radiat. Phys. Chem..

[4] El-Atwani O., Hinks J.A., Greaves G., Gonderman S., Qiu T., Efe M., Allain J.P. (2014). In-situ TEM observation of the response of ultrafine-and nanocrystalline-grained tungsten to extreme irradiation environments. Sci. Rep..

[5] Koch C.C., Morris D.G., Lu K., Inoue A. (1999). Ductility of Nanostructured Materials. MRS Bull..

[6] Hohenwarter A., Pippan R. (2015). Fracture and fracture toughness of nanopolycrystalline metals produced by severe plastic deformation. Philos. Trans. Ser. A Math. Phys. Eng. Sci..

[7] Hall E.O. (1951). The Deformation and Ageing of Mild Steel: III Discussion of Results. Proc. Phys. Soc. B.

[8] Petch N.J. (1953). The cleavage strength of polycrystals. J. Iron Steel Inst..

[9] Armstrong R.W. (2014). 60 Years of Hall-Petch: Past to Present Nano-Scale Connections. Mater. Trans..

[10] Kormout K.S., Pippan R., Bachmaier A. (2017). Deformation-Induced Supersaturation in Immiscible Material Systems during High-Pressure Torsion. Adv. Eng. Mater..

[11] Moszner F., Cancellieri C., Chiodi M., Yoon S., Ariosa D., Janczak-Rusch J., Jeurgens L. (2016). Thermal stability of Cu/W nano-multilayers. Acta Mater..

[12] Zhao M., Schlueter K., Wurmshuber M., Reitgruber M., Kiener D. (2021). Open-cell tungsten nanofoams: Scaling behavior and structural disorder dependence of Young’s modulus and flow strength. Mater. Design.

[13] Raghavan R., Wheeler J.M., Esqué-de los Ojos D., Thomas K., Almandoz E., Fuentes G.G., Michler J. (2015). Mechanical behavior of Cu/TiN multilayers at ambient and elevated temperatures: Stress-assisted diffusion of Cu. Mater. Sci. Eng. A.

[14] Raghavan R., Wheeler J.M., Harzer T.P., Chawla V., Djaziri S., Thomas K., Philippi B., Kirchlechner C., Jaya B.N., Wehrs J. (2015). Transition from shear to stress-assisted diffusion of copper–chromium nanolayered thin films at elevated temperatures. Acta Mater..

[15] Wheeler J.M., Raghavan R., Chawla V., Zechner J., Utke I., Michler J. (2015). Failure mechanisms in metal–metal nanolaminates at elevated temperatures: Microcompression of Cu–W multilayers. Scr. Mater..

[16] Zhao M., Pfeifenberger M.J., Kiener D. (2020). Open-cell tungsten nanofoams: Chloride ion induced structure modification and mechanical behavior. Results Phys..

[17] Allen F.I., Hosemann P., Balooch M. (2020). Key mechanistic features of swelling and blistering of helium-ion-irradiated tungsten. Scr. Mater..

[18] El-Atwani O., Suslova A., Novakowski T.J., Hattar K., Efe M., Harilal S.S., Hassanein A. (2015). In-situ TEM/heavy ion irradiation on ultrafine-and nanocrystalline-grained tungsten: Effect of 3 MeV Si, Cu and W ions. Mater. Charact..

[19] Juarez T., Biener J., Weissmüller J., Hodge A.M. (2017). Nanoporous Metals with Structural Hierarchy: A Review. Adv. Eng. Mater..

[20] Scheiber D., Pippan R., Puschnig P., Ruban A., Romaner L. (2016). Ab-initio search for cohesion-enhancing solute elements at grain boundaries in molybdenum and tungsten. Int. J. Refract. Met. Hard Mater..

[21] Hohenwarter A., Bachmaier A., Gludovatz B., Scheriau S., Pippan R. (2009). Technical parameters affecting grain refinement by high pressure torsion. Int. J. Mater. Res..

[22] Vüllers F., Spolenak R. (2015). From solid solutions to fully phase separated interpenetrating networks in sputter deposited “immiscible” W–Cu thin films. Acta Mater..

[23] Pfeifenberger M.J., Mangang M., Wurster S., Reiser J., Hohenwarter A., Pfleging W., Kiener D., Pippan R. (2017). The use of femtosecond laser ablation as a novel tool for rapid micro-mechanical sample preparation. Mater. Design.

[24] Lucas B.N., Oliver W.C. (1999). Indentation power-law creep of high-purity indium. Met. Mater. Trans. A.

[25] Oliver W.C., Pharr G.M. (1992). An improved technique for determining hardness and elastic modulus using load and displacement sensing indentation experiments. J. Mater. Res..

[26] Maier V., Durst K., Mueller J., Backes B., Höppel H.W., Göken M. (2011). Nanoindentation strain-rate jump tests for determining the local strain-rate sensitivity in nanocrystalline Ni and ultrafine-grained Al. J. Mater. Res..

[27] Korte S., Stearn R.J., Wheeler J.M., Clegg W.J. (2012). High temperature microcompression and nanoindentation in vacuum. J. Mater. Res..

[28] Kappacher J., Renk O., Kiener D., Clemens H., Maier-Kiener V. (2021). Controlling the high temperature deformation behavior and thermal stability of ultra-fine-grained W by re alloying. J. Mater. Res..

[29] Liang L., Li M., Qin F., Wei Y. (2013). Temperature effect on elastic modulus of thin films and nanocrystals. Philos. Mag..

[30] Milman Y., Golubenko A., Dub S.N. (2011). Indentation size effect in nanohardness. Acta Mater..

[31] Chang Y.A., Himmel L. (1966). Temperature Dependence of the Elastic Constants of Cu, Ag, and Au above Room Temperature. J. Appl. Phys..

[32] Lowrie R., Gonas A.M. (1965). Dynamic Elastic Properties of Polycrystalline Tungsten, 24°–1800 °C. J. Appl. Phys..

[33] Auciello O., Chevacharoenkul S., Ameen M.S., Duarte J. (1991). Controlled ion beam sputter deposition of W/Cu/W layered films for microelectronic applications. J. Vac. Sci. Technol. A Vac. Surf. Film..

[34] Sun Y., Ren Y., Yang K. (2016). New preparation method of micron porous copper through physical vacuum dealloying of Cu–Zn alloys. Mater. Lett..

[35] Alcock C.B., Itkin V.P., Horrigan M.K. (1984). Vapour Pressure Equations for the Metallic Elements: 298–2500 K. Can. Metall. Q..

[36] Willard Gibbs J. (1874). On the Equilibrium of Heterogeneous Substances.

[37] Wulff G. (1901). Zur Frage der Geschwindigkeit des Wachsthums und der Auflösung der Krystallflächen. Z. Kristallogr. Cryst. Mater..

[38] Barmparis G.D., Lodziana Z., Lopez N., Remediakis I.N. (2015). Nanoparticle shapes by using Wulff constructions and first-principles calculations. Beilstein J. Nanotechnol..

[39] Wang Y., Wang Z., Dinh C.-T., Li J., Ozden A., Golam Kibria M., Seifitokaldani A., Tan C.-S., Gabardo C.M., Luo M. (2020). Catalyst synthesis under CO2 electroreduction favours faceting and promotes renewable fuels electrosynthesis. Nat. Catal..

[40] Hansen P.L., Wagner J.B., Helveg S., Rostrup-Nielsen J.R., Clausen B.S., Topsøe H. (2002). Atom-resolved imaging of dynamic shape changes in supported copper nanocrystals. Science.

[41] Kappacher J., Leitner A., Kiener D., Clemens H., Maier-Kiener V. (2020). Thermally activated deformation mechanisms and solid solution softening in W-Re alloys investigated via high temperature nanoindentation. Mater. Design.

[42] Monclús M.A., Karlik M., Callisti M., Frutos E., Llorca J., Polcar T., Molina-Aldareguía J.M. (2014). Microstructure and mechanical properties of physical vapor deposited Cu/W nanoscale multilayers: Influence of layer thickness and temperature. Thin Solid Film..

[43] Gröger R., Vitek V. (2009). Temperature and strain rate dependent flow criterion for bcc transition metals based on atomistic analysis of dislocation glide. Int. J. Mater. Res..

[44] Maier V., Hohenwarter A., Pippan R., Kiener D. (2015). Thermally activated deformation processes in body-centered cubic Cr—How microstructure influences strain-rate sensitivity. Scr. Mater..

[45] Li J., Lu B., Zhang Y., Zhou H., Hu G., Xia R. (2020). Nanoindentation response of nanocrystalline copper via molecular dynamics: Grain-size effect. Mater. Chem. Phys..

[46] Huang C.-C., Chiang T.-C., Fang T.-H. (2015). Grain size effect on indentation of nanocrystalline copper. Appl. Surf. Sci..

[47] Schiøtz J., Jacobsen K.W. (2003). A maximum in the strength of nanocrystalline copper. Science.

[48] Kamaya M. (2009). A procedure for estimating Young’s modulus of textured polycrystalline materials. Int. J. Solids Struct..

[49] Kim H.S., Hong S.I., Kim S.J. (2001). On the rule of mixtures for predicting the mechanical properties of composites with homogeneously distributed soft and hard particles. J. Mater. Process. Technol..

[50] Simmons G., Wang H. (1971). Single Crystal Elastic Constants and Calculated Aggregate Properties: A Handbook.

[51] Shen T.D., Koch C.C., Tsui T.Y., Pharr G.M. (1995). On the elastic moduli of nanocrystalline Fe, Cu, Ni, and Cu–Ni alloys prepared by mechanical milling/alloying. J. Mater. Res..

[52] Wei Q., Jiao T., Ramesh K., Ma E., Kecskes L., Magness L., Doeding R., Kazykhanov V., Valiev R. (2006). Mechanical behavior and dynamic failure of high-strength ultrafine grained tungsten under uniaxial compression. Acta Mater..

[53] Wei Q., Cheng S., Ramesh K., Ma E. (2004). Effect of nanocrystalline and ultrafine grain sizes on the strain rate sensitivity and activation volume: Fcc versus bcc metals. Mater. Sci. Eng. A.

[54] Maier V., Schunk C., Göken M., Durst K. (2015). Microstructure-dependent deformation behaviour of bcc-metals—Indentation size effect and strain rate sensitivity. Philos. Mag..

[55] Fukuda M., Tabata T., Hasegawa A., Nogami S., Muroga T. (2016). Strain rate dependence of tensile properties of tungsten alloys for plasma-facing components in fusion reactors. Fus. Eng. Des..

[56] Chen J., Lu L., Lu K. (2006). Hardness and strain rate sensitivity of nanocrystalline Cu. Scr. Mater..

[57] Zhu T., Li J., Samanta A., Kim H.G., Suresh S. (2007). Interfacial plasticity governs strain rate sensitivity and ductility in nanostructured metals. Proc. Natl. Acad. Sci. USA.

[58] Kiener D., Fritz R., Alfreider M., Leitner A., Pippan R., Maier-Kiener V. (2019). Rate limiting deformation mechanisms of bcc metals in confined volumes. Acta Mater..

[59] Wang Y., HAMZA A., Ma E. (2006). Temperature-dependent strain rate sensitivity and activation volume of nanocrystalline Ni. Acta Mater..

[60] Suo T., Li Y., Xie K., Zhao F., Zhang K.-S., Deng Q. (2011). Experimental investigation on strain rate sensitivity of ultra-fine grained copper at elevated temperatures. Mech. Mater..

[61] Asaro R.J., Suresh S. (2005). Mechanistic models for the activation volume and rate sensitivity in metals with nanocrystalline grains and nano-scale twins. Acta Mater..

[62] Fritz R., Wimler D., Leitner A., Maier-Kiener V., Kiener D. (2017). Dominating deformation mechanisms in ultrafine-grained chromium across length scales and temperatures. Acta Mater..

