# Supplementary material for: High-Temperature Nanoindentation of an Advanced Nano-Crystalline W/Cu Composite
Source: Nanomaterials (Basel). 2021 Nov 3;11(11):2951. doi: 10.3390/nano11112951 (PMC8618606; doi:10.3390/nano11112951)
Supplement: Supplementary file 1 [file nanomaterials-11-02951-s001.zip › nanomaterials-1437462-supplementary.pdf]

Supplementary:

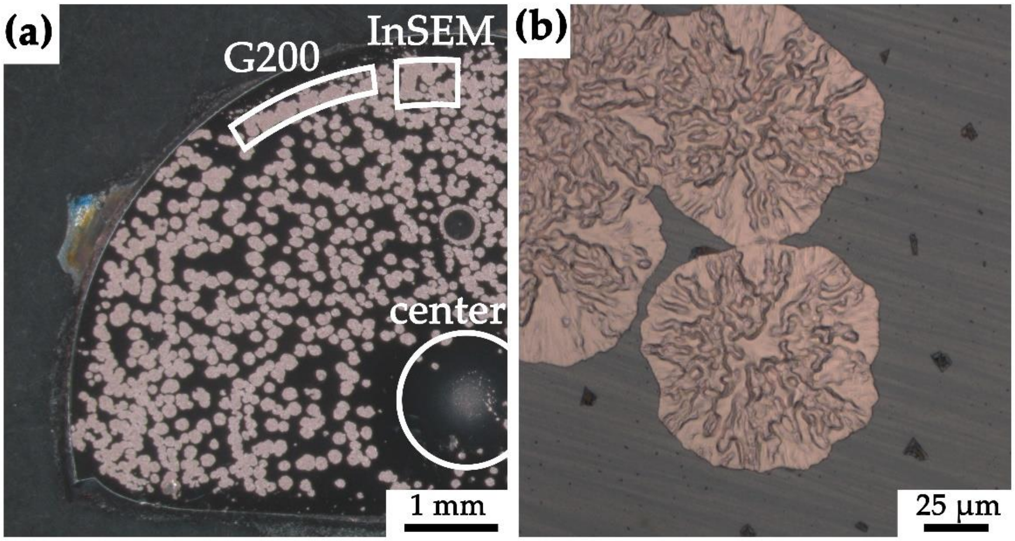

S1: Macroscopic light optical microscopic images of the surface of a high-pressure deformed W/Cu nc composite disc. The specimen was heat-treated at 300 °C within a vacuum furnace for 1h and subsequently tested at several temperatures up to 600 °C by nanoindentation. In (**a**), the regions within the white rectangles represent the tested areas during high-temperature nanoindentation tests. Here, an accumulation of Cu pools on the surface, near and overgrowing the indentation sites, indicating a deformation-induced formation of them, is visible. In the center region, the applied deformation was not sufficient to evoke Cu pool formation. In (**b**), Cu pools next to and overgrowing indents are displayed.
